# Scalable and heterogenous mobile robot fleet-based task automation in crowded hospital environments—a field test

**DOI:** 10.3389/frobt.2022.922835

**Published:** 2022-08-23

**Authors:** Robert Valner, Houman Masnavi, Igor Rybalskii, Rauno Põlluäär, Erik Kõiv, Alvo Aabloo, Karl Kruusamäe, Arun Kumar Singh

**Affiliations:** Intelligent Materials and Systems Lab, Institute of Technology, University of Tartu, Tartu, Estonia

**Keywords:** robot fleet, ROS, healthcare, human-robot interaction, mobile robotics, object transportation, RMF

## Abstract

In hospitals, trained medical staff are often, in addition to performing complex procedures, spending valuable time on secondary tasks such as transporting samples and medical equipment; or even guiding patients and visitors around the premises. If these non-medical tasks were automated by deploying mobile service robots, more time can be focused on treating patients or allowing well-deserved rest for the potentially overworked healthcare professionals. Automating such tasks requires a human-aware robotic mobility system that can among other things navigate the hallways of the hospital; predictively avoid collisions with humans and other dynamic obstacles; coordinate task distribution and area coverage within a fleet of robots and other IoT devices; and interact with the staff, patients and visitors in an intuitive way. This work presents the results, lessons-learned and the source code of deploying a heterogeneous mobile robot fleet at the Tartu University Hospital, performing object transportation tasks in areas of intense crowd movement and narrow hallways. The primary use-case is defined as transporting time-critical samples from an intensive care unit to the hospital lab. Our work builds upon Robotics Middleware Framework (RMF), an open source, actively growing and highly capable fleet management platform which is yet to reach full maturity. Thus this paper demonstrates and validates the real-world deployment of RMF in an hospital setting and describes the integration efforts.

## 1 Introduction

In hospitals, trained medical staff are often spending valuable time on secondary tasks such as transporting medicine and laboratory samples [Kriegel et al. (2021)]. If these non-medical tasks were automated by deploying mobile service robots [Evans (1994); Takahashi et al. (2010); Fragapane et al. (2020); Nambiappan et al. (2022)], more time can be focused on treating patients or giving well-deserved rest for the potentially overworked healthcare professionals. The demand for automating transportation has been identified as the main requirement for alleviating the workload of healthcare professionals to free up time from secondary, non-professional duties [Kriegel et al. (2021)]. Despite the positive sentiment [Marín et al. (2021)] emerging from the rise of robotic offerings, there are still a multitude of challenges—technical and human factors—that need to be addressed before the true benefits of autonomous mobile systems are achieved (Tornbjerg et al. (2021)).

Automating transportation tasks requires autonomous human-aware robotic mobility system that can among other things navigate the hallways of the hospital [Evans (1994)]; predictively avoid collisions with humans and other dynamic obstacles [Zheng et al. (2021); Kruse et al. (2013)]; coordinate task distribution and area coverage within a fleet of robots and other IoT devices [Ortiz et al. (2021)]; and interact with the staff, patients and visitors in an intuitive way [Ahn et al. (2019); Nambiappan et al. (2022)]. In order to discover and highlight the opportunities and limitations associated with the integration of mobile robots to the hospital workflow, further development, deployment, and validation is required for full systems that incorporate the previously listed capabilities in a scalable and extendable fashion.

We present the results, lessons learned and the source code of deploying a heterogeneous mobile robot fleet for transporting time-critical samples from an intensive care unit to the laboratory at the Tartu University Hospital. These autonomous mobile robots navigate narrow hallways with intense crowd movement. Our developed system showcases the level of maturity for several technological capabilities and tools. Among them, the Robotics Middleware Framework (RMF) [Quigley (2020)], an open-source fleet management system for interoperability of heterogeneous robotic systems.

## 2 System overview

This work focuses on automating the delivery of objects across the premises of a hospital. The primary use-case is defined as transporting time-critical samples, e.g., blood samples, from an intensive care unit to the hospital lab. This section will first provide a detailed overview of the hospital environment and associated challenges, followed by the description of an openly available software framework for robotic fleet management, the RMF, used as a basis for our particular use case. All our application-specific source code, configuration files and setup instructions are openly available via GitHub (organization page^1^; replication package^2^).

### 2.1 Hospital environment

**FIGURE 1:**
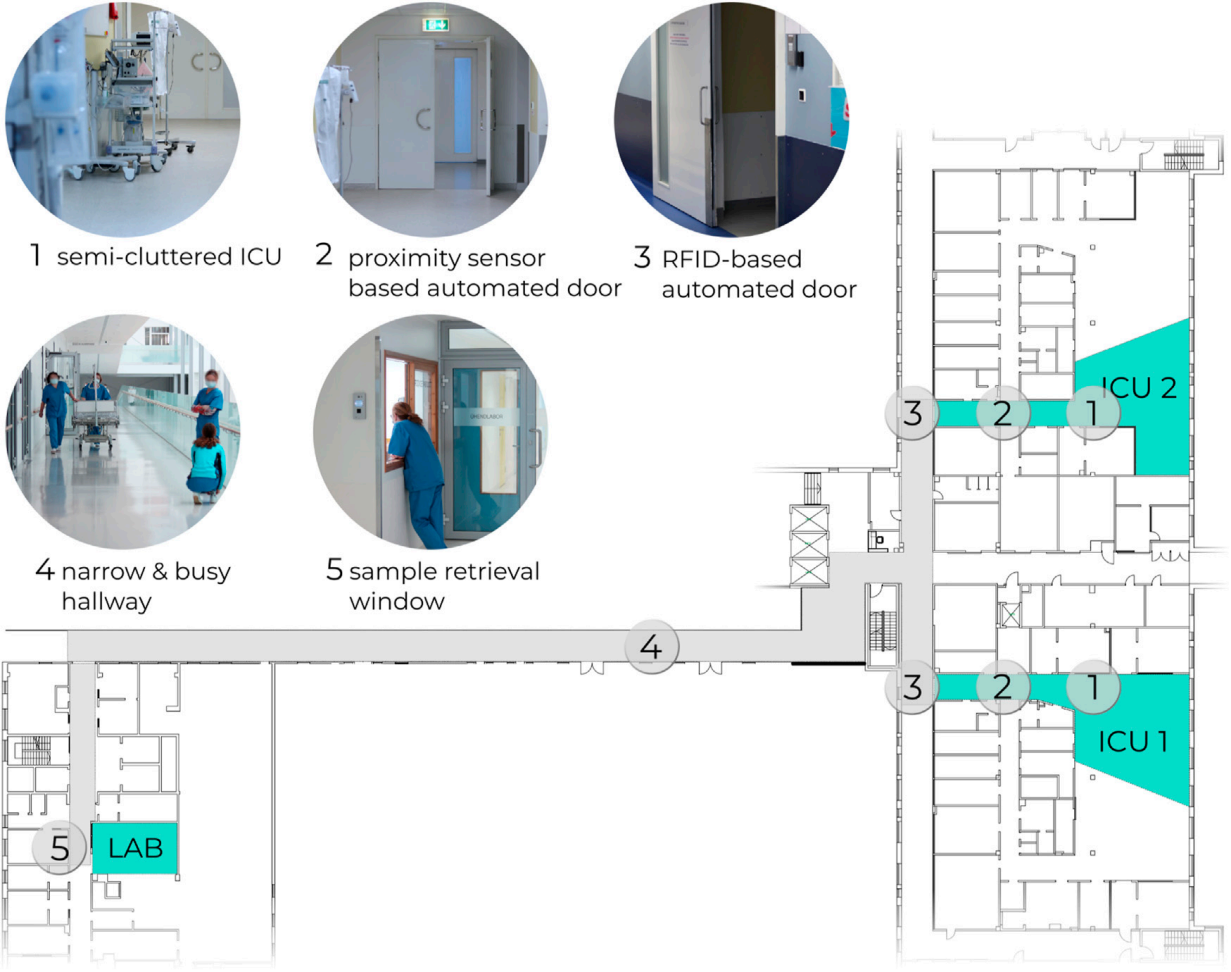
Floorplan of the section in Tartu University Hospital, where the object transportation tasks were deployed.

Figure 1 shows the floorplan of the work area along with key strategic sections. The sample transportation takes place between the Intensive Care Unit (ICU) and the lab. Along the path the robot is challenged by:

• Going through two semi-automated doors.
• Avoiding collisions with objects and people in cluttered areas and in the narrow and busy hallway.
• Placing the samples in the retrieval window.

The semi-automated doors require a triggering signal to open. Both doors are placed between the ICU and the hallway, forming a buffer zone. The door which opens to the hallway is operated via RFID tag. The other door, which opens to the ICU, is operated via a close-range proximity sensor, commonly triggered by waving a hand close by. Since there is no central automation server for the doors, the existing infrastructure had to be retrofitted with controller units (Section 2.4) that open the doors programmatically.

### 2.2 Robotics middleware framework

**FIGURE 2:**
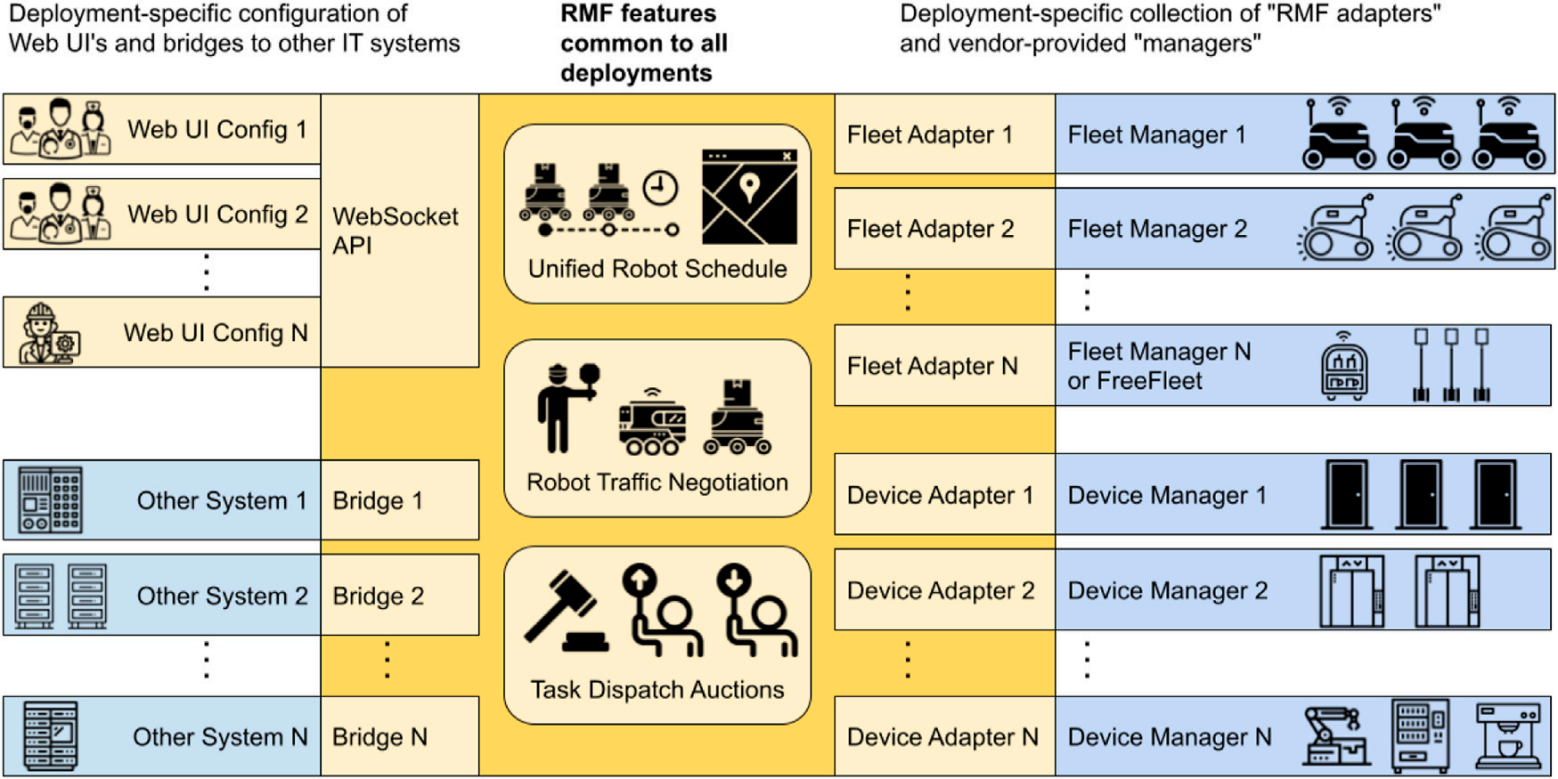
Run-time architecture of Robotics Middleware Framework Quigley (2020).

RMF [Quigley (2020)] (Figure 2) is an openly available framework of software tools and standards, with the goal of standardizing the integration and control of heterogeneous robot fleets and in-building infrastructure such as elevators and doors. The main emphasis of RMF is on scalability both in terms of integrating robotic fleets from different vendors, and managing a growing fleet, i.e., avoiding traffic congestions and giving way to robots with a high priority task. Despite the significant amount of robotic fleet management efforts reported in the literature [Ortiz et al. (2021); Thiel et al. (2009); Thamrongaphichartkul et al. (2020)], RMF uniquely remains as an actively maintained open-source and scalable fleet management system with a growing user community.

RMF contains run-time tools that orchestrate the robotic fleet and the infrastructure (Figure 2) as well as design-time tools for streamlining the configuration of RMF. The run-time system is structured as a distributed application based on ROS2. Task scheduling, traffic and infrastructure management, task distribution, and system visualization is segregated into individual ROS2 nodes which communicate via ROS2 messages. The communication between the RMF and robots, as well as building infrastructure, is achieved via adapters or fleet and device adapters specifically. Thus an adapter is a bridge that allows RMF to monitor and control a custom or proprietary robotic fleet (or infrastructure elements) via standard Application Programming Interface (API). RMF run-time accepts commands via ROS2 services and messages, allowing the developer to create custom user interfaces via Python, JavaScript or C++. By design RMF manages robots on fleet level (i.e., can simultaneously control multiple fleets from different vendors), not on the level of an individual robot. Thus for the robots that have no dedicated fleet manager system (custom-built robots, etc), FreeFleet can be used to allow RMF control such robots.

**FIGURE 3:**
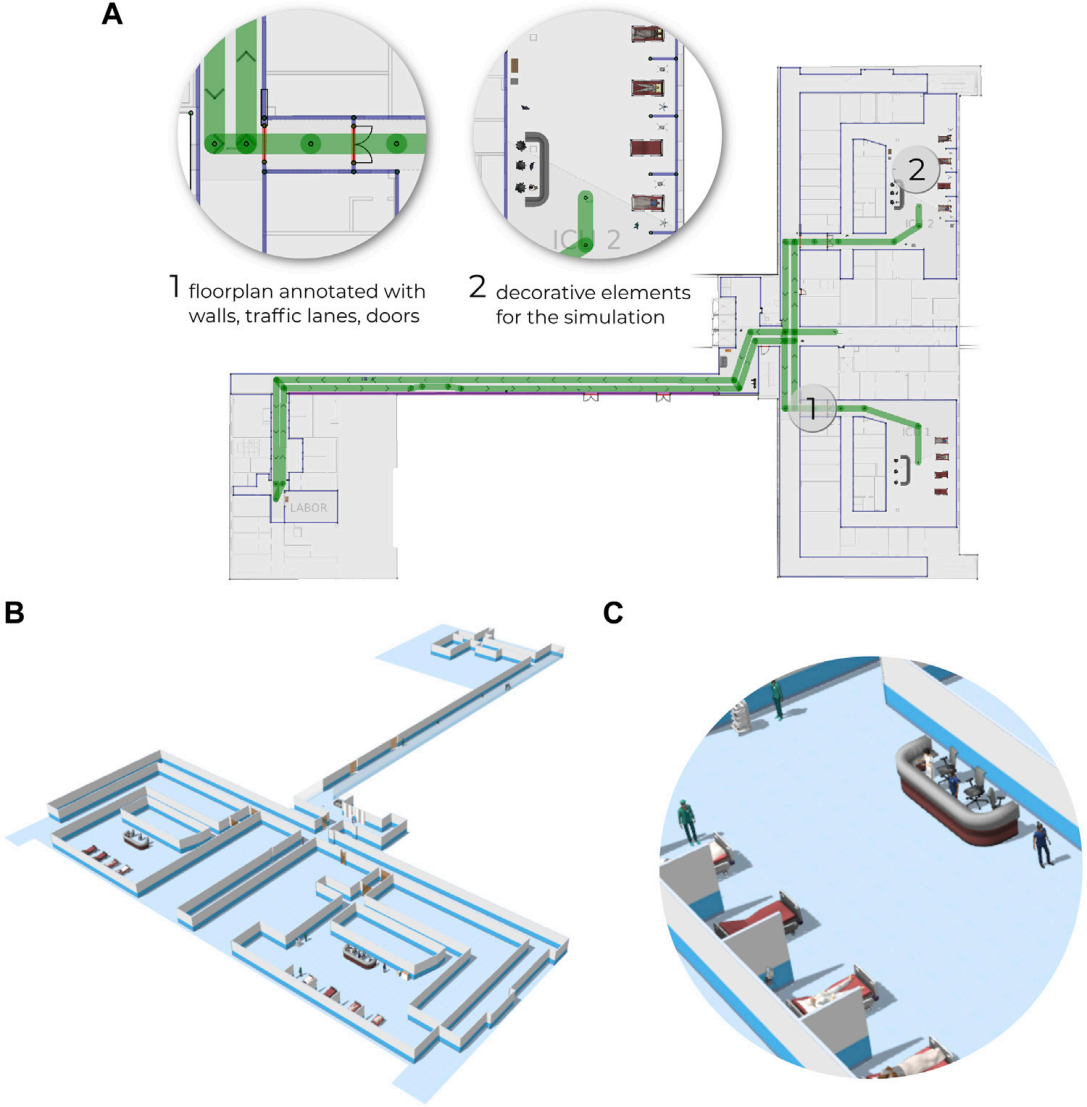
Hospital environment annotated via Traffic Editor **(A)** which is used to generate configuration files necessary for the RMF run-time and also to generate a simulated environment **(B)** containing furniture, people, etc **(C)**.

Traffic Editor is a graphical design-time tool, allowing developers to annotate the environment via defining walls, doors, floors, traffic lanes, charging points, strategic locations, etc (Figure 3A). Additionally decorative elements can be added, such as furniture, food dispensers, medical equipment, and static people. The annotated map is then used to generate the configuration files necessary for the RMF run-time, as well as a simulation environment (Figures 3B,C) which can be used to test the RMF configuration.

**FIGURE 4:**
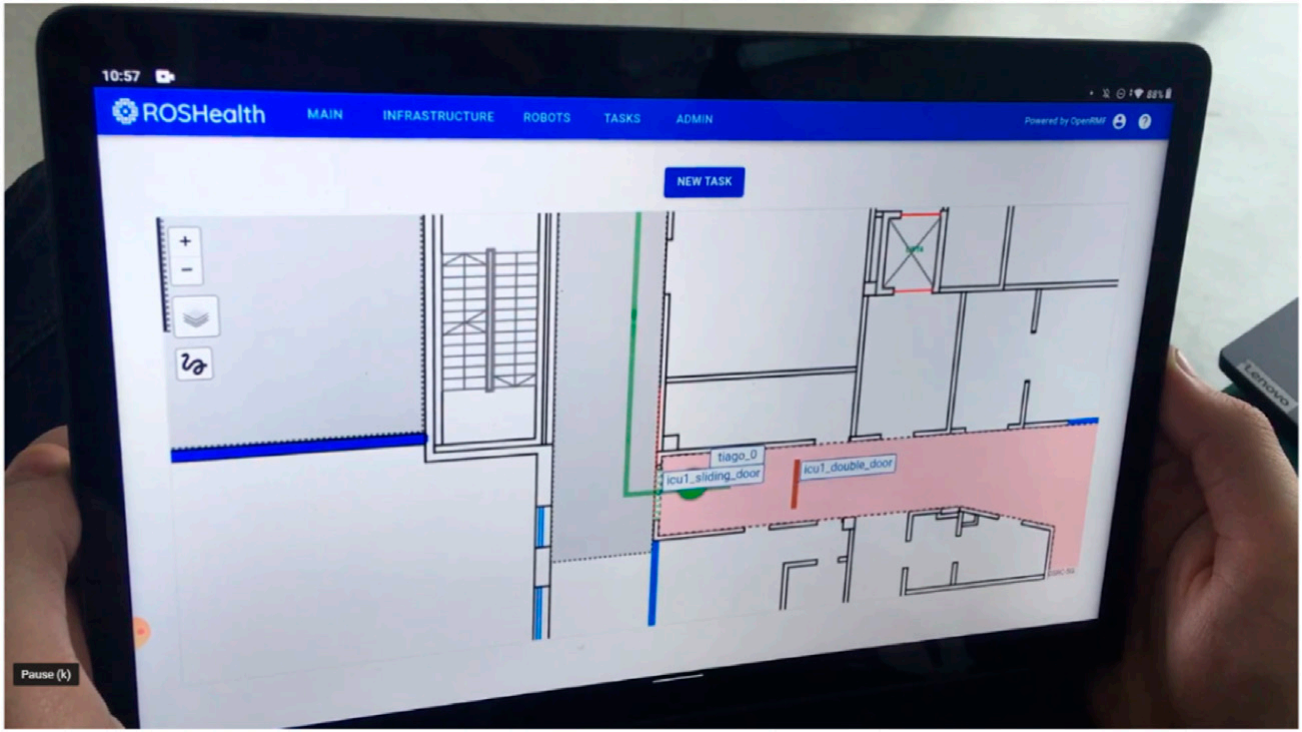
RMF Web is a web browser-based tool used to monitor and control the RMF connected system.

RMF Web (Figure 4) is a browser based tool which allows to monitor and control the RMF connected system, including opening/closing doors. RMF Web has a component-based structure, allowing developers to add customized interfaces while utilizing the existing tools of RMF Web.

RMF is rapidly developing with a growing user community, yet RMF still needs more testing and deployment in real-world environments in order to achieve a certain degree of reliability and maturity. Thus this work is a test-case of RMF in a real-world hospital, complete with logistics, infrastructure and safety related challenges. The next subsections describe the specific setup used to deploy RMF in the Tartu University Hospital.

### 2.3 Architecture

**FIGURE 5:**
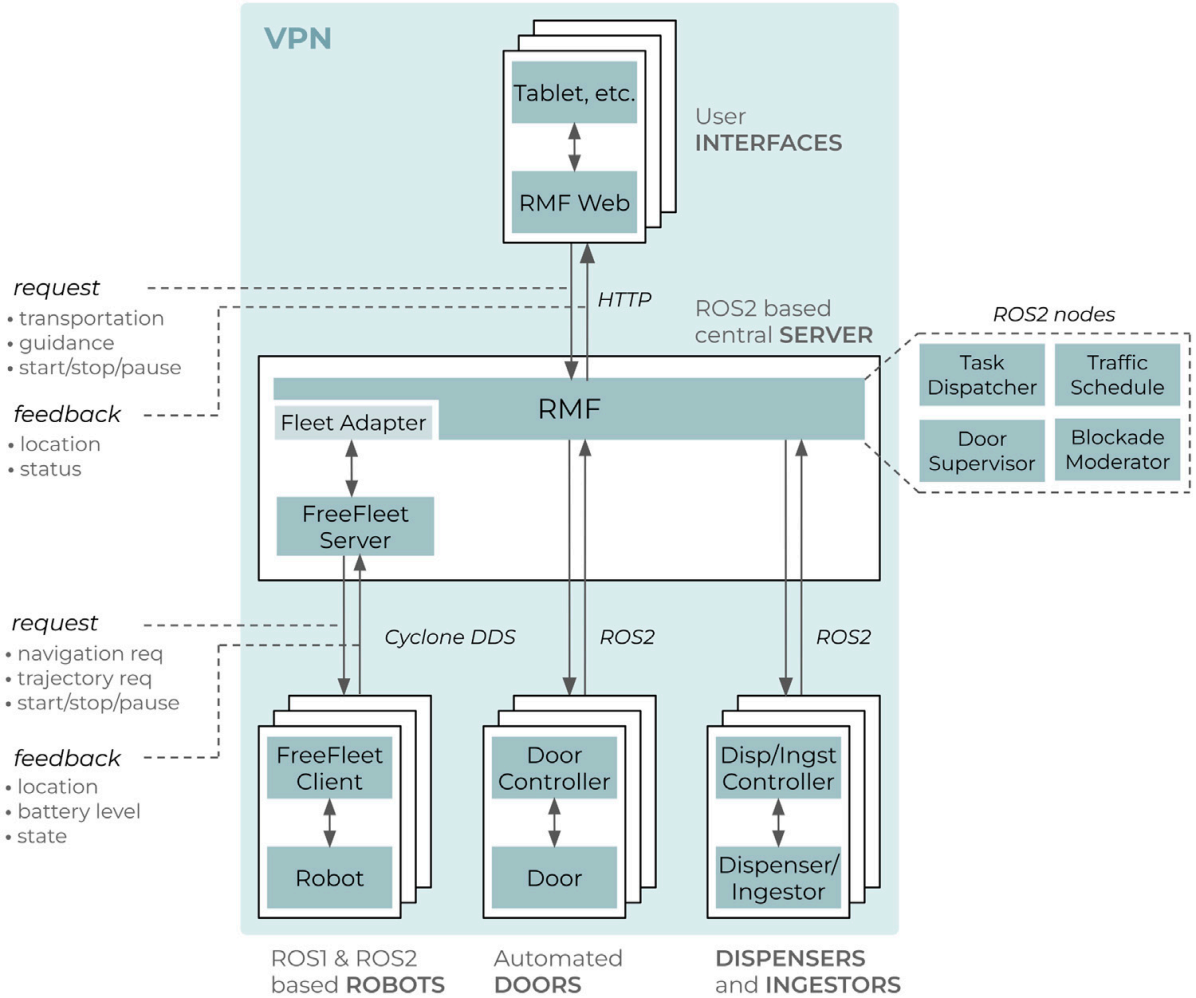
High-level architecture of the deployed system.

Figure 5 shows the architecture of our deployed system. The setup consists of robots; automated doors; user interface devices, such as tablets; dispensers and ingestors, which are used to place and retrieve objects from the robot respectively; and RMF, orchestrating the whole setup. All devices are connected via ZeroTier Virtual Private Network (VPN). Since the deployed robots had no dedicated fleet manager, we adopted FreeFleet. Structurally FreeFleet is segregated to a central server and clients, a client per each robot. The server is responsible for mediating navigation requests from RMF and providing feedback to RMF via aggregating the state of the whole fleet. A fleet adapter defines the characteristics of a robot within the fleet (e.g., battery capacity, acceleration, speed, footprint size) which is utilized by RMF for traffic scheduling. The FreeFleet client passes navigation requests from the server to the driver of the local robot (ROS Navigation) and provides feedback of the robot’s state. Both RMF and the FreeFleet server are running on a dedicated PC. FreeFleet server and clients communicate purely via Cyclone DDS, a Data Distribution Service (DDS) vendor, thus the server can simultaneously manage both ROS1 and ROS2 based robots. Each door, dispenser and ingestor is equipped with a custom controller (Section 2.4) that accepts commands from and sends feedback to RMF. Finally, RMF Web is utilized as a graphical user interface, mostly deployed on tablets via a web browser.

### 2.4 Hardware and software setup

While RMF provides a platform for integrating the whole system, each individual device, i.e. robots, doors, dispensers, ingestors, need a custom setup for bridging with RMF. In our use-case, both dispensing and ingesting procedures were accomplished by the user manually placing (dispense) and retrieving (ingest) the samples from the robot and verifying via single-button GUI on the touchscreen mounted to the robot. Technically samples can be transported without incorporating RMF’s dispensing and ingesting mechanism, but doing so allows RMF to see if the robot is involved in a dispensing or ingesting sequence, i.e., unavailable.

**FIGURE 6:**
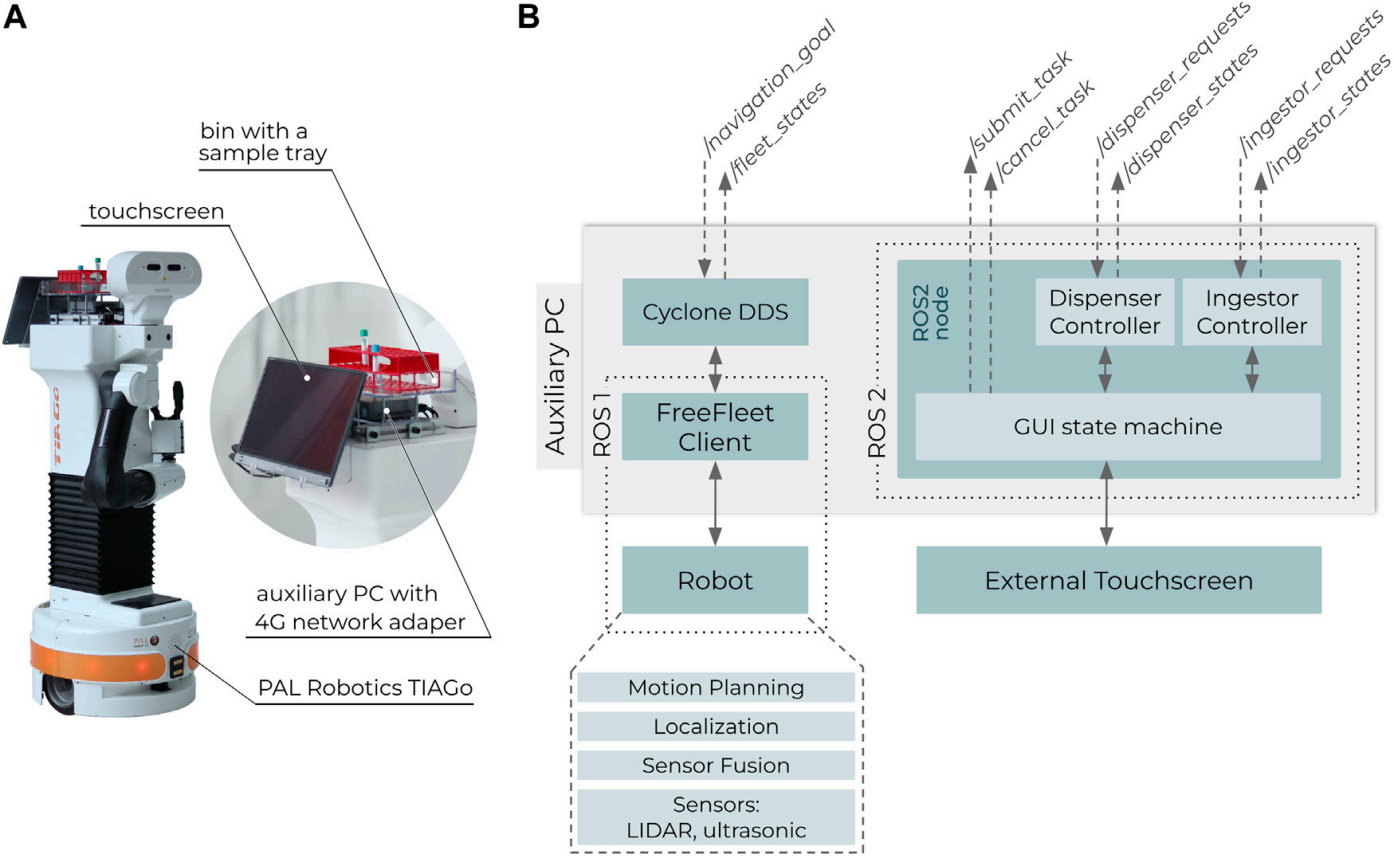
PAL Robotics TIAGo with additional hardware for the object transportation task **(A)**. Software setup of the whole robot **(B)**.

Figure 6 shows the hardware (Figure 6A) and software (Figure 6B) setup of the deployed robot PAL Robotics TIAGo in its factory software and hardware configuration. The robot was only customized by mounting a bin for carrying the sample trays; a touchscreen (ASUS ZenScreen MB16A) for human-robot interaction (i.e., sending the robot to the lab and verifying the dispensing/ingesting sequences); and an auxiliary PC (Intel NUC with Ubuntu 20.04) with a 4G network adapter for managing RMF specific setup and controlling the touchscreen. The auxiliary PC had ROS Melodic and ROS2 Dashing installed, where the FreeFleet Client mediated RMF’s navigation goals to the robot via ROS1, while the touchscreen controller was deployed as a ROS2 node. Table 1 describes the touchscreen-based sample delivery procedure in detail. The hospital area was mapped via teleoperating TIAGo in mapping mode. The resulting map was aligned with the hospital’s floorplan in the Traffic Editor.

**TABLE 1:** Description of the touchscreen-based sample delivery control algorithm implemented in the auxiliary PC of the robot.

Step	Description
1	*User issues a transportation request via pressing the button on the touchscreen*
2	*A task request is sent to RMF, outlining the pickup (ICU) and drop-off (lab) locations as well as the names for dispensers/ingestors*
3	*RMF sends a dispensing request, causing the GUI to ask for the user’s confirmation*
4	*User presses the button on the touchscreen, confirming that all items are placed into the bin*
5	*The dispenser controller notifies RMF that dispensing is complete*
6	*RMF starts sending navigation goals to the robot via FreeFleet.*
7	*Robot reaches the lab*
8	*RMF sends an ingestion request, causing the GUI to inform the user to remove the items from the bin*
9	*User presses a button on the touchscreen, confirming that all items are removed from the bin*
10	*The ingestor controller notifies RMF that ingesting is complete*
11	*The touchscreen controller node sends a request to RMF to move the robot back to the ICU.*
12	*RMF starts sending navigation goals to the robot via FreeFleet.*
13	*Robot reaches the ICU.*

**FIGURE 7:**
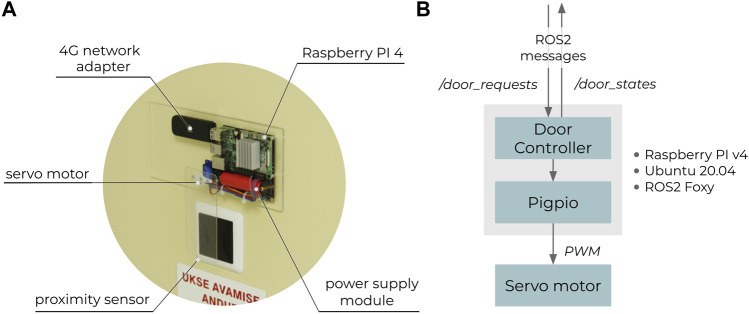
Custom hardware **(A)** for opening doors via RMF **(B)**.

Figure 7 shows the custom-made device for opening the semi-automated doors. Since the doors were operated either by a RFID card or a proximity sensor, a servo motor based system, which swipes the key card or triggers the proximity sensor, was designed for the initial system integration testing. The servo motor was either holding a key card (for RFID-based door) or a sheet of plastic (for proximity sensor based door). The servo motor was connected to a Raspberry PI 4, powered via a battery-based power supply module. A 4G network adapter was used to access the VPN (see Figure 5). The Raspberry had Ubuntu 20.04 and ROS2 Foxy installed. A door controller ROS2 node was created, which controlled the servo motor via PWM (hardware) when a request was received from RMF (“*/door_requests*” topic).

### 2.5 Scaling RMF fleet and deployed environments

This section describes the procedure of deploying the RMF setup in a new environment, as well as how new heterogeneous robots can be appended to the existing RMF fleet. The following chapters only cover the fundamental steps for a concise overview. Please refer to our online materials^3^ for a technical reference.

#### 2.5.1 Setting up a new environment

RMF is designed for static environments having a fixed number of walls, doors, elevators. Thus a floorplan of the environment is the basis for setting RMF up, followed by:

1) **Annotation**: The floorplan is annotated in the Traffic Editor by adding walls, traffic lanes, chargers, locations, doors, etc. Additional items, such as furniture or people, are added as the annotated map can be to generate a simulated environment.
2) **Mapping**: The physical environment is mapped with tools such as OpenSlam Gmapping (Grisetti et al., 2007) to generate a grid map. While RMF uses the annotated floorplan for controlling the system, each robot traditionally uses a grid map representation of the environment.
3) **Alignment**: The grid map is aligned with the floorplan inside the Traffic Editor. This is necessary for obtaining the coordinate transformation between the two map representations.
4) **Configuration**: ROS2 launch files are configured to start-up RMF with the appropriate environment and coordinate transformation obtained from the previous step. Examples can be seen in our online materials at GitHub.

#### 2.5.2 Setting up a new robot fleet

As RMF is based on ROS, any mobile robot supporting ROS1 or ROS2 with a Navigation^4^ setup can be controlled via RMF. If the fleet is homogeneous, then the setup and configurartion is identical to robots in the fleet. The following steps apply for robots without proprietary fleet management system, thus utilizing FreeFleet (described in Section 2.3):

1) **Mapping**: ROS-based robots require a grid map for navigation. An existing map can be reused or a new one created. This map can be shared with all members of the fleet.
2) **FreeFleet preparation**: Each robot is paired with a FreeFleet client application, which is normally installed on robot’s onboard computer. A FreeFleet server is also installed on the computer hosting RMF and normally one FreeFleet server instance is needed per homogeneous fleet.
3) **FreeFleet server configuration**: The name of the fleet is configured in the server along with the DDS configuration.
4) **RMF fleet adapter configuration**: RMF uses the characteristics of the robots within the fleet for traffic scheduling. These characteristic values are described in the RMF fleet adapter configuration file. Note that heterogeneous robots can technically be combined under single RMF fleet adapter and FreeFleet server, but RMF is then unaware of each robot’s individual technical characteristics, which can potentially lead to undefined behavior.
5) **FreeFleet client configuration**: If the robots’s ROS Navigation topic and action names are different from default values, FreeFleet client is configured for the correct names. The client is set-up with identical DDS configurations and a fleet name as the server.

## 3 Results

### 3.1 Demonstration

**FIGURE 8:**
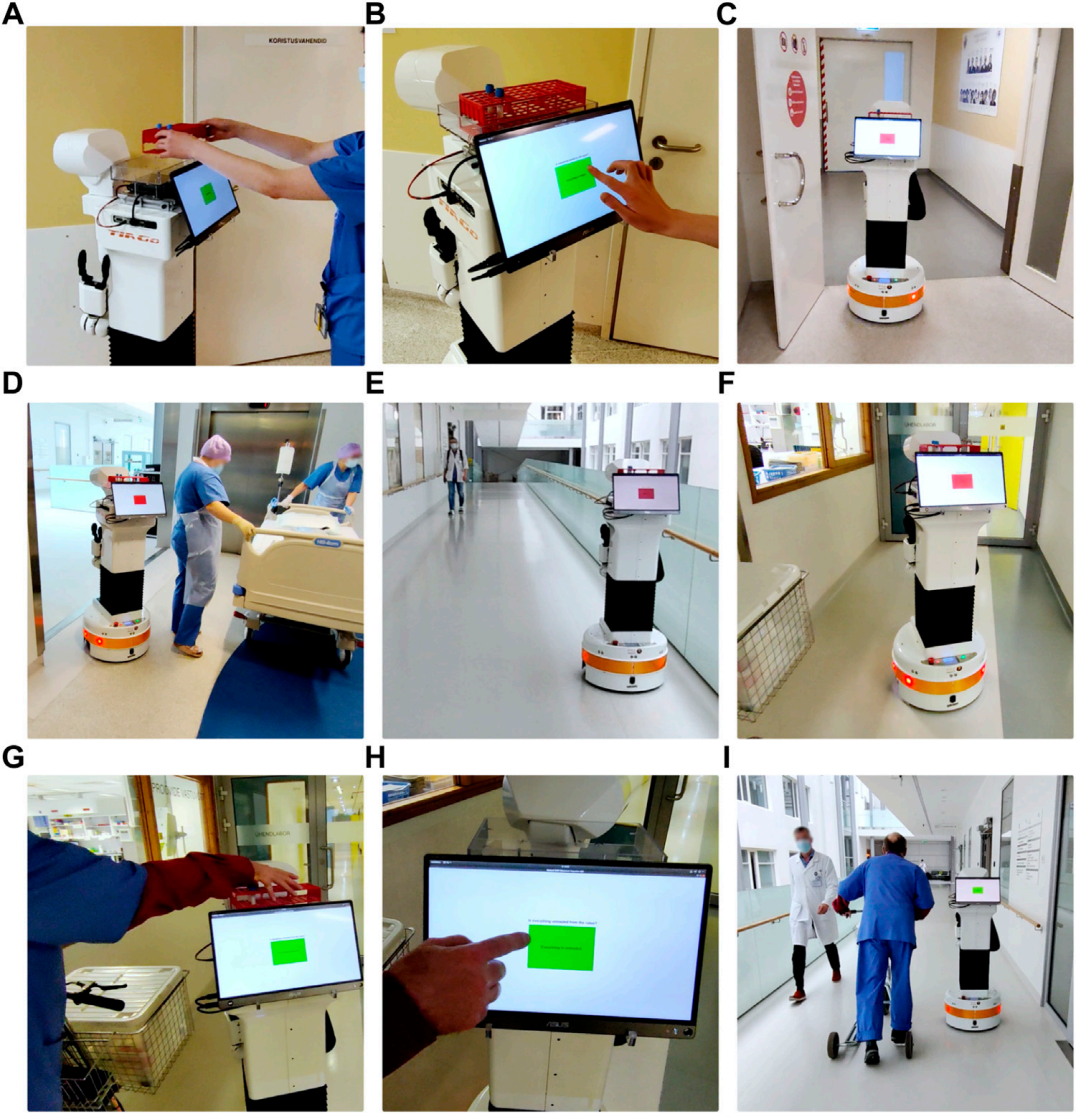
TIAGo performing a sample delivery run, initiated by a medical worker **(A)** followed by verification of the request **(B)**. Next, RMF started sending navigation goals to the robot and controlling the automated doors **(C)**. Avoiding dynamic obstacles **(D)** and traversing the hallway, keeping safely to the right **(E)**, the robot reached the lab **(F)** where a medical worker handed over the samples **(G)** and verified the delivery **(H)**. After the verification, the robot navigated back to the ICU, waiting for a new delivery request **(I)**.

The feasibility of this work was validated via a full scale deployment, where PAL Robotics TIAGo successfully transported blood samples from ICU to the lab (Figure 8). A video demonstrating one full run is available in the Supplementary Material along with technical feature demonstration of two heterogeneous robots controlled via RMF. The detailed step-by-step technical description of the delivery process is covered in Table 1. The sample delivery run was initiated by a medical worker placing the samples on the robot (Figure 8A) followed by verification of the request via the integrated touchscreen (Figure 8B). Next, RMF started sending navigation goals to the robot and controlling the automated doors (Figure 8C). The robot was able to avoid dynamic obstacles (Figure 8D) and traverse the hallway, keeping safely to the right (Figure 8E). Arriving at the lab (Figure 8F), a medical worker handed over the samples (Figure 8G) and verified that the samples were delivered (Figure 8H). After the verification, the robot navigated back to the ICU and remained idle, waiting for a new delivery request (Figure 8I).

### 3.2 Lessons learned and limitations

The successful deployment of this work is a result of thorough testing and validation, which has lead to accumulation of design and deployment related recommendations:

• **Use time optimally**—Test your system starting from simulation, then in a similar physical location and finally in the actual deployment area. This allows to maximize the valuable time spent on-site for testing and discovering new challenges, not debugging issues that could have been caught earlier.
• **Try to avoid reliance on public networks**—While the hospital had a public Wi-Fi available, using a 4G network had fewer technical constraints (e.g., more bandwidth, no firewalls, better signal coverage). However, it was crucial to test for any signal coverage dead zones.
• **Get DDS right**—ROS2 relies on DDS for communication. Make sure that your configuration is set up properly, e.g., supports multicast communication and all nodes have the same DDS configuration.
• **Double-check the navigation maps**—Mapping large areas will likely result in skewed maps due to the cumulative uncertainty of state estimation. A more accurate map can be obtained by mapping the area in smaller sections and merging the sub-maps manually with an image processing software. Also maps should be carefully reviewed to remove any artifacts (e.g., occupied cells in the middle of the hallway) that might result in undesired navigation behavior.
• **Stay out of the way**—Make sure that the robot spends a minimal amount of time in the vicinity of the elevators and as close to the walls as it can, as the hospital’s traffic is very intense and time critical. Use a two single direction traffic lane layout wherever possible.

RMF is very flexible and scalable in terms of task domains and device integration, but real world comes with a fair share of open challenges:

• **Waiting out congestions**—Narrow hallways get congested and in such situations it would be best for the robot to wait in a safe area until the congestion is cleared. However this would require a) a way to detect congestion and b) a method of informing RMF about the potential blockade. At the moment RMF does not support such behavior.
• **Emergency stopping**—The medical staff must be able to halt the robot and get it out of the way. Thus there is a tradeoff between the bulkiness and the functionality of the robot. A well equipped mobile manipulator is functionally very capable but also potentially heavy.
• **Safe integration of legacy infrastructure**—Most hospitals do not have programmatically controllable elevators and doors. While we created a temporary solution for opening the doors (Section 2.4), it was surely not sustainable because the devices ran out of power and introduced a security threat both by exposing the RFID card and having no further cybersecurity measures other than a protected VPN. Yet we see this as a common challenge in all hospitals that utilize mobile robots. A semi-automated door can be modified, so that the custom module directly controls the door (via electric signal), but such modifications can be against local security policies.

## 4 Discussion

**FIGURE 9:**
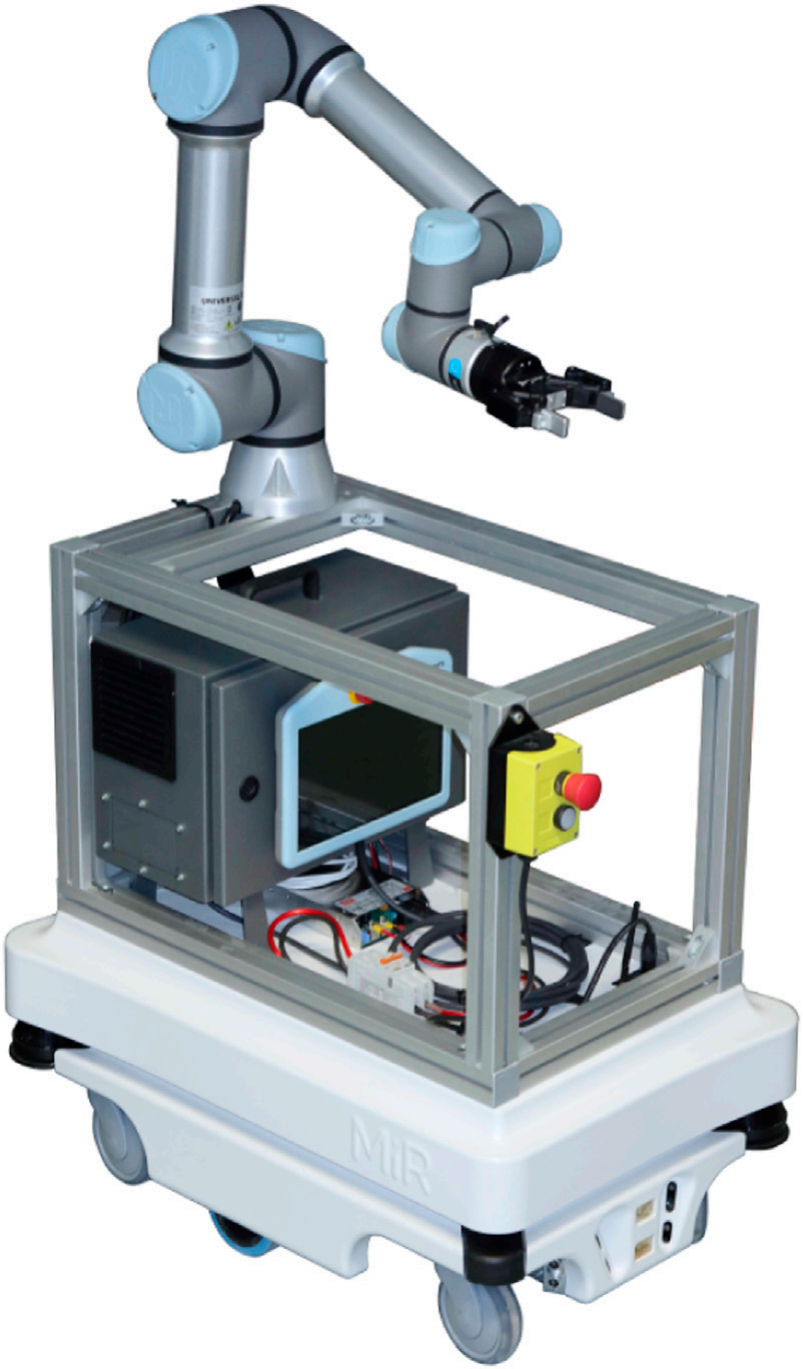
MiR100 mobile platform with UR5e manipulator. As a future work, we plan to utilize a mobile manipulator for sample handover, which is a necessary addition to the manual dispensing and ingesting setup.

This work demonstrated the feasibility of transporting objects with mobile robots, and utilizing the Robotics Middleware Framework as the core for coordinating robots and building’s infrastructure, such as doors. Our setup was validated in Tartu University Hospital, where PAL Robotics TIAGo transported blood samples from an active ICU to the lab through busy hallways. The ongoing and future work will focus on:

• **Tamper-proofing the transported samples**—Securing the samples into a closed container is essential when moving away from the testing and validation phase.
• **Automating the sample handover**—While the ICU staff does not mind placing the samples manually into the bin, the lab staff has to leave the lab in order to retrieve the samples from the robot. Utilizing a manipulator (mobile or static) for sample handover is a necessary addition to the manual dispensing and ingesting setup.
• **Utilizing an industrial mobile manipulator**—As a continuation of the previous point, we are transitioning from TIAGo to a mobile manipulator based on the MiR100 mobile platform and UR5e manipulator (Figure 9).
• **Increasing the number and diversity of robots**—While we have validated RMF’s capability to manage multiple heterogeneous robots (TIAGo and Clearpath Jackal mobile platforms) in a testing environment, we are yet to apply multiple delivery robots inside the Hospital, which would potentially increase the sample delivery throughput.
• **Covering more tasks**—Due to the peculiar layout of the Tartu University Hospital, people tend to get lost in its premises. The medical staff has admitted that during critical periods, guiding people can take up too much of valuable time. Thus we are actively developing a robot for guiding people around the hospital.

## Data Availability

Publicly available datasets were analyzed in this study. This data can be found here: GitHub organization page—https://github.com/scafld, Replication package—https://doi.org/10.5281/zenodo.6467038.

## Footnotes

1
https://github.com/scafld.

2
https://doi.org/10.5281/zenodo.6467038.

3
https://doi.org/10.5281/zenodo.6467038.

4
http://wiki.ros.org/navigation.
